# Preoperative muscle thickness influences muscle activation after arthroscopic knee surgery

**DOI:** 10.1007/s00167-021-06820-4

**Published:** 2021-12-18

**Authors:** Jorge Amestoy, Daniel Pérez-Prieto, Raúl Torres-Claramunt, Juan Francisco Sánchez-Soler, Albert Solano, Joan Leal-Blanquet, Pedro Hinarejos, Joan Carles Monllau

**Affiliations:** 1 Hospital del Mar, Barcelona, Spain; 2 Autonomous University of Barcelona, Barcelona, Spain

**Keywords:** Knee arthroscopy, Quadriceps muscle activation, Quadriceps muscle atrophy, Patellofemoral pain, Quadriceps muscle strength

## Abstract

**Purpose:**

The aim of this study was to compare the correlation between preoperative quadriceps femoris muscle thickness and postoperative neuromuscular activation and quadriceps femoris strength in patients with and without patellofemoral pain after arthroscopic partial meniscectomy.

**Methods:**

A series of 120 patients were prospectively analysed in a longitudinal cohort study of patients scheduled for arthroscopic partial meniscectomy. The patellofemoral pain group included patients who developed anterior knee pain after surgery while the control group included those who had not done so. Patients with preoperative patellofemoral pain, previous knee surgeries as well as those on whom additional surgical procedures had been performed were excluded. Of the 120 initially included in the study, 90 patients were analysed after the exclusions.

**Results:**

There is a direct correlation between preoperative quadriceps femoris muscle thickness and the neuromuscular activity values and the strength of the muscle at 6 weeks after surgery. These results were seen exclusively in the group of patients who do not develop patellofemoral pain (0.543, *p* = 0.008). The group of patients who developed anterior knee pain in the postoperative period did not show this correlation (n.s.).

**Conclusion:**

In patients without patellofemoral pain after meniscectomy, the greater the preoperative thickness of the quadriceps femoris, the more postoperative neuromuscular activation and strength they had. This correlation did not occur in those patients who develop patellofemoral pain after meniscal surgery.

**Level of evidence:**

II.

## Introduction

Quadriceps activation failure (QAF) occurs due to alterations in neural signalling caused by a reduction in alpha motor neuron pool recruitment and/or firing rate [12]. It commonly occurs after knee surgery and is not simply an isolated local phenomenon related to atrophy. This has been attributed to arthrogenic muscle inhibition, [21] a process in which quadriceps activation failure is caused by neural inhibition [30].

Activation failure is the inability to completely volitionally contract the muscle due to alterations in neural signalling. It is common following any type of knee surgery [12, 14]. If left untreated, QAF can significantly impede strength gains by only allowing portions of the muscles to be volitionally utilized during active exercise [13, 19].

If these neural abnormalities are not targeted with specific interventions used to disinhibit an inhibited muscle, quadriceps dysfunction may persist and become a factor limiting successful postoperative knee management [17, 19].

Quadriceps muscle hypotrophy (QMH) that occurs following knee surgery is also thought to contribute to persistent muscle weakness [17, 39] due to alterations in muscle architecture [23], selective fibre atrophy [20, 21], or even neural deficits like QAF [25]. It might cause patellofemoral pain (PFP), a dreaded complication after knee surgery. It affects up to 23% of patients who undergo arthroscopic partial meniscectomy (APM) [1, 5].

Muscle hypotrophy as well as the delayed onset of electromyographic activity of the quadriceps femoris muscle after arthroscopic partial meniscectomy predispose to the development of postoperative PFP. Furthermore, these two risk factors also predispose to worse postoperative functional results [1].

Despite the important role that the quadriceps muscle plays in this pathology, whether having greater quadriceps muscle thickness before surgery has any impact in the neuromuscular activation of this muscle in the postoperative period has not yet been studied. No prospective study investigating the development of patellofemoral pain after a knee arthroscopy has tested the electromyographic activity of the vastus medialis and vastus lateralis muscles and its relationship with the preoperative muscular thickness.

The aim of this study was to compare the correlation between preoperative quadriceps muscle thickness, its postoperative neuromuscular activation and strength in patients with and without patellofemoral pain after APM.

The hypothesis was that there is direct correlation between the preoperative quadriceps muscle thickness and its neuromuscular activity after an APM in patients who do not develop patellofemoral pain.

## Materials and methods

Approval for the study was granted by the Ethics Committee of Clinical Research of Parc de Salut Mar Hospital, Autonomous University of Barcelona (CEIC no. 2014/5534). Between 2015 and 2017, a prospective longitudinal cohort study was carried out on consecutive patients who were scheduled to undergo APM. The inclusion criteria were that the patient be aged 18 years or older and have an acute symptomatic medial meniscal tear requiring surgery. All patients underwent the procedure at a maximum of 6 months of evolution from the meniscal tear. No differences were found in the time of evolution of the meniscal tear between the groups. The exclusion criteria included having had PFP prior to surgery, previous surgeries on the involved knee (including meniscal repair) or if there had been an associated surgical procedure (e.g. chondral repair, ACL reconstruction, etc.) during the index procedure.

**Fig. 1:**
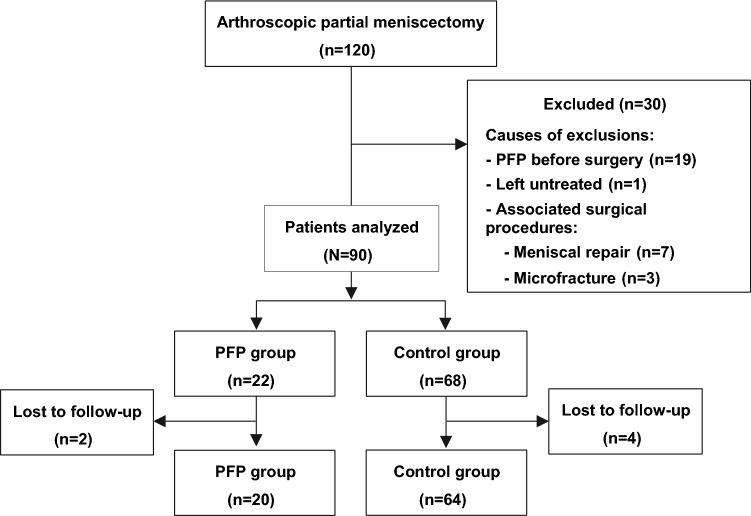
Flowchart of the study and enrolment of the patients. PFP, patellofemoral pain

For the reasons previously stated, 30 patients out of the 120 initially included in the study were excluded. Nineteen of the 30 had had PFP before surgery. The remaining 11 patients of those 30 had undergone an associated surgical procedure like meniscal repair (7), microfractures due to the incidental presence of a chondral injury (3), and there was 1 partial meniscal injury that was left untreated (Fig. 1).

### Surgical procedure

The same team of knee surgeons from Parc de Salut Mar Hospital carried out all the surgical procedures on the patients. They were done with the patients under spinal anaesthesia (15 mg levobupivacaine 0.5%). As the surgery was of short duration, a tourniquet was used at a pressure of 100 mmHg above systolic pressure with prior exsanguination of the limb. In all cases, the arthroscopy was performed through routine anterolateral and anteromedial portals. None of the patients had a femoral or a sciatic nerve block subsequent to the procedure. No drains were used in any case.

### Postoperative management

The patients underwent the operation on a day-case basis. The same anti-inflammatory and anticoagulant medication was given to all the patients during the postoperative period. All patients passed through a physical therapy program either in our institution or in external rehabilitation facilities after discharge. In both cases, the same postoperative guidelines were respected. It was a standardized physical therapy protocol based on immediate postoperative weight-bearing with crutches as tolerated, without bracing, until there was a normal gait pattern. Range-of-motion was not limited and progressed as tolerated.

The progressive program that the patients followed included strengthening, proprioception and coordination and cardiovascular exercises. The program included targeted strengthening exercises for the lower extremity muscles (quadriceps, hamstrings, hip and calf muscles). It went from isometric exercises to open chain exercises over 6 weeks. For the first 3–4 weeks after surgery, knee flexion during weight-bearing exercises (e.g. squats, lunges) was limited to 60º. Exercise intensity, of a maximum of one repetition, ranged between 65 and 80% and the volume was three sets of 12–20 repetitions. With a focus on neuromuscular control of the operated knee, proprioception and coordination exercises included moderate intensity tasks (e.g. single-leg balance and static and dynamic stabilization drills on stable and unstable surfaces for 10 min/sessions). The 10-min cardiovascular exercise session called for cycling at light-to-moderate intensity. All patients received the same standardized physical therapy protocol after the surgery.

### Outcome assessment

Patients were assigned to a group depending on how they responded to a question relative to the presence of PFP at the preoperative visit and at 6 weeks after surgery (“Have you ever had pain in the anterior part of the knee in addition to the current pain on the medial or lateral joint line?”). The question was then answered in writing by the patient.

To quantify VM and VL muscle thickness, magnetic resonance imaging (MRI) of the thigh was taken for all patients before surgery and at 6 weeks after surgery. Those MRIs were done on both the injured knee and the contralateral knee. A high correlation coefficient exists between the quadriceps cross-sectional area and the total muscle volume [22]. The knees were imaged on the sagittal plane on the same 1.5T whole-body MRI unit (GE SIGNA EXCITE) using a commercial receive-only extremity coil. A topogram was taken and axial planes were programmed in a T1 fast spin-echo 2D sequence (flip angle 55°, repetition time 580 ms, minimum TE time 11.30 ms, field of view 17 × 17 cm, 60 partitions, 448 × 288 pixel matrix, acquisition time 2.55 min). Sagittal images were obtained at a partition thickness of 6 mm with a partition interval of 4.50 mm and an in-plane resolution of 0.31–0.83 mm. All the MRI assessments were blinded to subject identification, time sequences and other knee structural measurements. This measurement was performed at 3.75 cm for the VM and 15 cm for the VM and VL from the upper pole of the patella, in accordance with Wang et al. [37]. The VL/VM ratio was calculated with those values [26]. All of the MRI measurements were performed by two blinded radiologists, specialized in musculoskeletal system, as independent observers.

Likewise, the electrical contractility of the quadriceps femoris was analysed with surface electromyography (S-EMG) (MEGAWIN), extracting muscle activity and the maximum voluntary contraction values of the VL and the VM during the preoperative period and at 6 weeks after surgery. Four Ag/AgCl surface electrodes (30 mm diameter) were distributed in the direction of the muscle fibres of the VM and VL, in accordance with the method for electrode placement in lower limb muscles S-EMG recordings described by Rainoldi et al. [28]. Two additional control electrodes were placed on the bone surface of medial and lateral tibial plateau. A 95% alcohol solution was used to clean the skin under the electrodes.

Patients were first informed about with electrical stimulation. Then, a single current intensity of 1-ms rectangular pulses was progressively increased in 10-mA steps (starting from 0 mA) every 3 to 5 s. Maximal current intensity was determined as the current level at which the evoked torque did not further increase despite increasing current intensity, indicating full quadriceps recruitment. Subsequently, the patients executed a standardized warm-up protocol consisting of 6 submaximal voluntary contractions and 1 maximum voluntary contraction (MVC) with 90 degrees of knee flexion. Next, patients completed 3 MVC trials separated by approximately 30 s. Standardized verbal encouragement and visual feedback were consistently provided to the patients. MVC torque was measured as the peak torque adjusted to body mass attained before or after the superimposed twitch [27]. The activation level was calculated using the following formula: [100 − (superimposed twitch torque/potentiated twitch torque) × 100]. [38]

An isokinetic test (Biodex dynamometer) was also performed both before surgery and 6 weeks after surgery to assess the muscle performance values. The tests provided data on muscular strength through range of motion at 60 degrees per second. The patients did the extension, with passive return to the starting position. Three repetitions were performed, and the median was chosen for each patient. The electrophysiological and isokinetic tests were performed on both knees by the same physiotherapist who was blinded as to whether the patient had patellofemoral pain.

### Statistical analysis

Numerical variables are expressed descriptively as mean and standard deviations. Within the groups, changes (preoperative vs. postoperative) were evaluated by means of paired *t* tests. This was performed separately for the PFP group and the control group. The correlation between continuous variables were evaluated with Spearman’s rank correlation coefficients. STATA version 15.10 (StataCorp, College Station, TX, USA) was used for the statistical analysis. *p* values of 0.05 were considered statistically significant.

A sample size calculation was made beforehand. Accepting an alpha risk of 0.05, a beta risk of 0.20 and a relative risk greater or equal to 0.10, 88 subjects were needed. The proportion of patients who developed PFP after surgery was estimated to be 0.25, the same as the incidence in healthy people [29, 31, 34]. A follow-up loss of 5% was assumed. The Poisson approximation was used.

Results

Of the remaining 90 patients after exclusion, 6 were lost to follow-up. The losses included 4 patients from the control group and 2 patients from PFP group. These follow-up losses were found to be non-differential for the statistical analysis of the data, because they did not affect the demographics of the two groups. Of the remaining 84 patients, 20 (23.8%) were allocated to PFP group for developing postoperative anterior knee pain, and 64 patients (76.2%) were considered controls. The mean age of the sample was 44.9 years (SD 11.0 years). There were 29 women (34.5%) and 55 men (65.5%). Both groups were comparable in terms of all the preoperative variables analysed (Table 1).

There is a moderate positive correlation between preoperative quadriceps muscle thickness and preoperative muscle activity, MVC and strength values, regardless of whether the patient develops patellofemoral pain or not. This correlation is stronger for the VM at 3.75 cm of the patella (0.6, *p* < 0.01) (Tables 1, 2, 3, 4). A moderate to high positive correlation exists between preoperative quadriceps femoris muscle thickness and muscle thickness at 6 weeks after surgery. This correlation is independent of whether the patient develops patellofemoral pain or not (Table 2).

**Table 1:** Study variables analysed

	PFP group (*n* = 20)	Control group (*n* = 64)	*p* value
Preoperative			
VL 15 cm (cm^2^)	21.1 ± 3.6	22.3 ± 3.7	n.s
VM 15 cm (cm^2^)	15.8 ± 2.9	17.2 ± 2.7	n.s
VM 3.75 cm (cm^2^)	17.7 ± 2.6	19.2 ± 4.0	n.s
VL MA (µV)	2418.3 ± 940.9	2686.0 ± 984.8	n.s
VM MA (µV)	2477.1 ± 936.3	2626.9 ± 914.4	n.s
VL MVC (µV)	266.9 ± 70.8	264.4 ± 115.5	n.s
VM MVC (µV)	271.2 ± 80.7	248.9 ± 109.2	n.s
MS 60 degrees per s (Kg)	23.6 ± 8.6	25.1 ± 9.2	n.s
Postoperative			
VL 15 cm (cm^2^)	15.9 ± 2.5	20.7 ± 3.3	** < 0.01**
VM 15 cm (cm^2^)	9.0 ± 2.2	14.9 ± 2.9	** < 0.01**
VM 3.75 cm (cm^2^)	9.7 ± 1.8	16.6 ± 3.9	** < 0.01**
VL MA (µV)	1614.0 ± 671.7	2199.1 ± 840.2	**0.02**
VM MA (µV)	1226.3 ± 565.8	1946.1 ± 799.3	** < 0.01**
VL MVC (µV)	159.8 ± 55.9	222.3 ± 63.3	**0.04**
VM MVC (µV)	122.9 ± 63.9	231.8 ± 62.8	** < 0.01**
MS 60 degrees per s (Kg)	12.27 ± 5.6	20.0 ± 5.9	** < 0.01**

Data are reported as mean ± SD. Bold *p* values indicate a statistically significant difference between groups (*p* < 0.05). *VL* vastus lateralis, *VM* vastus medialis, *VMO* vastus medialis oblique, *MA* muscle activity, *MVC* maximum voluntary contraction, *MS* muscular strength

**Table 2:** Postoperative correlations

Preoperative	Control group (*n* = 64)		
	VL 15 cm	VM 15 cm	VM 3.75 cm
**Preoperative**			
VL MA	0.4 **(*p* = 0.01**)	0.3 **(*p* = 0.01**)	0.2 (***p* = 0.03**)
VM MA	0.3 (*p* = n.s.)	0.4 **(*p* = 0.08**)	0.6 **(*p* < 0.01**)
VL MVC	0.4 (***p* = 0.03**)	0.2 (*p* = n.s.)	0.3 (***p* = 0.01**)
VM MVC	0.2 **(*p* = 0.03**)	0.4 (***p* = 0.03**)	0.4 (***p* < 0.01**)
MS 60 degrees per s	0.5 (***p* = 0.04**)	0.4 (***p* = 0.01**)	0.4 (***p* = 0.02**)
**Postoperative**			
VL 15 cm	0.7 (***p* < 0.01**)	0.3 (***p* = 0.01**)	0.5 (***p* < 0.01**)
VM 15 cm	0.2 (***p* = 0.04**)	0.8 (***p* < 0.01**)	0.4 (***p* < 0.01**)
VM 3.75 cm	0.6 (*p* = n.s.)	0.4 (***p* = 0.01**)	0.7 (***p* < 0.01**)
VL MA	0.6 (***p* = 0.04**)	0.1 (*p* = n.s.)	0.3 (***p* = 0.03**)
VM MA	0.3 (*p* = n.s.)	0.7 **(*p* = 0.04**)	0.5 (***p* = 0.01**)
VL MVC	0.5 (***p* < 0.01**)	0.4 (***p* = 0.02**)	0.3 (***p* = 0.04**)
VM MVC	0.2 **(*p* = 0.03**)	0.3 (*p* = n.s.)	0.5 (***p* = 0.02**)
MS 60 degrees per s	0.4 (***p* = 0.02**)	0.4 (***p* = 0.01**)	0.5 (***p* = 0.01**)

Preoperative	PFP group (*n* = 20)		
	VL 15 cm	VM 15 cm	VM 3.75 cm
**Preoperative**			
VL MA	0.3 (***p* = 0.04**)	0.1 (*p* = n.s.)	0.1 (*p* = n.s.)
VM MA	0.3 (*p* = n.s.)	0.3 **(*p* = 0.04**)	0.4 (***p* = 0.02**)
VL MVC	0.3 (***p* = 0.04**)	0.1 (*p* = n.s.)	0.3 (*p* = n.s.)
VM MVC	0.2 (*p* = n.s.)	0.3 (*p* = n.s.)	0.0 (*p* = n.s.)
MS 60 degrees per s	0.1 (*p* = n.s.)	0.4 (***p* = 0.03**)	0.1 (*p* = n.s.)
**Postoperative**			
VL 15 cm	0.5 (***p* = 0.02**)	0.5 (***p* = 0.04**)	0.5 (***p* < 0.01**)
VM 15 cm	0.23(***p* = 0.04**)	0.6 (***p* < 0.01**)	0.4 (***p* = 0.04**)
VMO or VM 3.75 cm	0.4 (*p* = n.s.)	0.6 (***p* = 0.01**)	0.6 (***p* < 0.01**)
VL MA	0.1 (*p* = n.s.)	0.2 (*p* = n.s.)	0.1 (*p* = n.s.)
VM MA	0.1 (*p* = n.s.)	0.1 (*p* = n.s.)	0.2 (*p* = n.s.)
VL MVC	0.1 (*p* = n.s.)	0.1 (*p* = n.s.)	0.2 (*p* = n.s.)
VM MVC	0.1 (*p* = n.s.)	0.1 (*p* = n.s.)	0.1 (*p* = n.s.)
MS 60 degrees per s	0.1 (*p* = n.s.)	0.5 (*p* = n.s.)	0.1 (*p* = n.s.)

Data are reported as mean ± SD. Bold *p* values indicate a statistically significant difference between groups (*p* < 0.05). *VL* vastus lateralis, *VM* vastus medialis, *VMO* vastus medialis oblique, *MA* muscle activity, *MVC* maximum voluntary contraction, *MS* muscular strength

**Table 3:** Postop correlations

Postoperative	Control group (*n* = 64)		
	VL 15 cm	VM 15 cm	VM 3.75 cm
**Postoperative**			
VL MA	0.5 (***p* = 0.01**)	0.2 (*p* = n.s.)	0.3 (***p* = 0.03**)
VM MA	0.2 (***p* = 0.03**)	0.5 (***p* = 0.04**)	0.5 (***p* = 0.04**)
VL MVC	0.4 (***p* < 0.01**)	0.2 **(*p* < 0.01**)	0.2 (***p* < 0.01**)
VM MVC	0.2 (*p* = n.s.)	0.3 (*p* = n.s.)	0.5 (***p* = 0.04**)
MS 60 degrees per s	0.3 (***p* = 0.02**)	0.4 (***p* = 0.01**)	0.5 (***p* = 0.03**)

Data are reported as mean ± SD. Bold *p* values indicate a statistically significant difference between groups (*p* < 0.05). *VL* vastus lateralis, *VM* vastus medialis, *VMO* vastus medialis oblique, *MA* muscle activity, *MVC* maximum voluntary contraction, *MS* muscular strength

**Table 4:** Postop correlations

Postoperative	PFP group (*n* = 20)		
	VL 15 cm	VM 15 cm	VM 3.75 cm
Postoperative			
VL MA	0.1 (*p* = n.s.)	0.3 (*p* = n.s.)	0.3 (*p* = n.s.)
VM MA	0.1 (*p* = n.s.)	0.2 (*p* = n.s.)	0.1 (*p* = n.s.)
VL MVC	0.1 (*p* = n.s.)	0.2 (*p* = n.s.)	0.2 (*p* = n.s.)
VM MVC	0.0 (*p* = n.s.)	0.1 (*p* = n.s.)	0.2 (*p* = n.s.)
MS 60 degrees per s	0.2 (***p* < 0.01**)	0.1 (*p* = n.s.)	0.3 (*p* = n.s.)

Data are reported as mean ± SD. Bold *p* values indicate a statistically significant difference between groups (*p* < 0.05). *VL* vastus lateralis, *VM* vastus medialis, *VMO* vastus medialis oblique, *MA* muscle activity, *MVC* maximum voluntary contraction, *MS* muscular strength

There was a moderate positive correlation between preoperative quadriceps femoris muscle thickness and muscle activity, MVC and strength values at 6 weeks after surgery exclusively for the group of patients who do not develop patellofemoral pain. The group of patients who developed patellofemoral pain in the postoperative period did not show this correlation (Table 2, 3, 4).

## Discussion

The most important finding of the current investigation is that while a positive correlation exists between preoperative quadriceps femoris thickness and postoperative neuromuscular activation in patients without patellofemoral pain, this does not occur in those patients who develop patellofemoral pain after meniscal surgery. The results suggest that the delayed onset of electromyographic activity of the vastus lateralis and especially the vastus medialis muscle, regardless of muscle thickness prior to surgery, could be considered as a risk factor for the development of patellofemoral pain around the sixth week after APM.

It is likely that there are a wide range of factors involved in the aetiology of anterior knee pain [10, 32, 33]. Muscle atrophy as well as the delay in the activation of the quadriceps femoris muscle have already been identified as risk factors for developing patellofemoral pain after arthroscopic knee surgery [1]. Quadriceps femoris muscle thickness has been decreased between 25 and 50% in the PFP group. Moreover, all the other measurement decreased remarkably compared to the control group.

The results indicate that patients in whom PFP appears after arthroscopic surgery experience muscular atrophy of the VL and, to a greater extent, the VM during a period of 6 weeks. This decrease in quadriceps femoris muscle size is probably related to postoperative proximity inhibition and the consequent failure of muscle activation. Then again, it might also be related to the development of PFP. This article goes further, as it shows the association between quadriceps activation failure and postoperative patellofemoral pain, even in those patients with good muscle thickness and good electrical contractility of the quadriceps muscles prior to surgery. In this line, QAF and QMH are crucial factors to target to improve the recovery of knee function following knee arthroscopy.

Another interesting finding of the current work is the incidence of PFP after an arthroscopic meniscectomy in patients who did not previously have this pain. The 23.8% incidence of postoperative PFP is similar to that of patients after ACL reconstruction at the 1- and 2-year follow-up (24% and 22%, respectively) [5]. However, the shorter follow-up time in the present investigation impedes drawing any firm conclusion with regard to this particular issue.

Recently, research has focused on developing specific disinhibitory interventions to improve voluntary quadriceps activation. Neuromuscular electrical stimulation has been shown to improve quadriceps function and strength, as well as decrease its atrophy in the postoperative period of ACL surgery [15, 19]. Eccentric exercise, whereby the muscle is lengthened and an external force exceeds that produced by the muscle, has been shown to be more effective than traditional concentric strengthening at minimizing muscle atrophy and improving muscle force production [9]. The ability to eccentrically contract the quadriceps is critical for optimal knee range of motion during the weight-acceptance phase of gait [11, 36], which is necessary in the early phase of rehabilitation after meniscal surgery [2, 3, 16, 18]. The combination of neuromuscular electrical stimulation with eccentric exercises in the postoperative rehabilitation protocol after meniscal surgery may improve early activation of the quadriceps femoris muscle. Therefore, they may aid in preventing the development of anterior knee pain, even in those patients with poor quadriceps muscle thickness.

A threshold of 6 weeks was set for the measurements. It is the moment in which the incidence of patellofemoral pain increases after knee arthroscopy [1, 2]. In general terms, although the treatment must be individualized for each patient, sixth weeks is the time point from which the patient should be able to return to play after an APM [16]. It is at this point that the patient should be able to fully activate the quadriceps femoris muscle.

A progressive pre-habilitation program that is mainly focused on strengthening the quadriceps femoris of subjects who have undergone meniscal surgery leads to improved knee function in the postoperative period, in the same way as happens in patients who have undergone ACL reconstruction [6, 8, 15, 19, 24]. However, based on the current results, those patients who develop patellofemoral pain after arthroscopy do not show this correlation.

There are some limitations in the present study. One is the severity of meniscal damage and consequently the amount of meniscus removed at surgery, as they might have an impact on the degree of postoperative electrical contractility of the quadriceps femoris. Then, the definition of PFP, which is based on the presence of pain in the anterior part of the knee in a self-referral manner and not on more objective and specific measure score or patellofemoral questionnaires like the patellar diagnostic test (Felson). [35] Lower extremity structural anomalies on the transverse plane like increased femoral anteversion and lateral tibial torsion may contribute to patellofemoral malalignment and PFP must also be considered [4, 7]. These factors have not been analysed in the current study. Thus, that might be another limitation.

In the light of the current results, early activation of the quadriceps femoris after APM is particularly important for the prevention of postoperative patellofemoral pain, regardless of the quadriceps muscle thickness prior to the intervention.

This study provide insights on the influence of neuromuscular control on anterior knee pain and how the postoperative rehabilitation protocol after meniscectomy should be approach. Those results may in turn be useful in guiding rehabilitation efforts and guide daily clinical practice after knee arthroscopy.

## Conclusion

In patients without patellofemoral pain after meniscectomy, the thicker the preoperative quadriceps femoris, the more postoperative neuromuscular activation and strength they have. This correlation did not occur in those patients who develop patellofemoral pain after meniscal surgery.
